# Changes in Physical Activity among United Kingdom University Students Following the Implementation of Coronavirus Lockdown Measures

**DOI:** 10.3390/ijerph18062792

**Published:** 2021-03-10

**Authors:** Alice Wickersham, Ewan Carr, Ryan Hunt, Jordan P. Davis, Matthew Hotopf, Nicola T. Fear, Johnny Downs, Daniel Leightley

**Affiliations:** 1 Department of Psychological Medicine, King’s College London, London SE5 8AF, UK; matthew.hotopf@kcl.ac.uk (M.H.); daniel.leightley@kcl.ac.uk (D.L.); 2 Biostatistics & Health Informatics, King’s College London, London SE5 8AF, UK; ewan.carr@kcl.ac.uk; 3 King’s Sport, King’s College London, London SE1 1NP, UK; ryan.hunt@kcl.ac.uk; 4 USC Center for Artificial Intelligence in Society, Suzanne Dworak-Peck School of Social Work, University of Southern California, Los Angeles, CA 90015-2212, USA; jordanpd@usc.edu; 5 South London and Maudsley NHS Foundation Trust, London SE5 8AZ, UK; johnny.downs@kcl.ac.uk; 6 King’s Centre for Military Health Research and Academic Department of Military Mental Health, King’s College London, London SE5 9RJ, UK; nicola.t.fear@kcl.ac.uk; 7 Department of Child and Adolescent Psychiatry, King’s College London, London SE5 8AF, UK

**Keywords:** COVID-19, coronavirus, remote measurement technology, physical activity, longitudinal, university students, young adult, lockdown

## Abstract

Coronavirus disease (COVID-19) and resulting restrictions have significantly impacted physical activity levels. However, objectively measured changes in physical activity levels among UK university students during lockdown are understudied. Using data collected via remote measurement technology from a mobile physical activity tracker, this study aimed to describe the longitudinal trajectories of physical activity following the start of lockdown among students at a large UK university, and to investigate whether these trajectories varied according to age, gender, and ethnicity. Continuous physical activity data for steps walked per week (*n* = 730) and miles run per week (*n* = 264) were analysed over the first period of lockdown and subsequent restriction easing using negative binomial mixed models for repeated measures. Throughout the observation period, more steps were walked by males compared to females, and by White groups compared to all other ethnic groups combined. However, there was a gradual increase in the number of steps walked per week following the commencement of lockdown, irrespective of sociodemographic characteristics. For females only, there was a decrease in the number of miles run per week following lockdown. The long-term impact of the pandemic on physical health is unknown, but our results highlight changes in physical activity which could have implications for physical health.

## 1. Introduction

In the United Kingdom (UK), a nationwide lockdown across England, Scotland, Wales and Northern Ireland was announced on 23 March 2020 in response to the severe acute respiratory syndrome coronavirus 2 (SARS-CoV-2) which causes COVID-19 [1]. Many services, including fitness centres, hospitality, leisure, and educational institutions were forced to close for an unspecified amount of time. Guidelines were issued by the UK Government stipulating that people should only go outside for one form of exercise per day or to make essential shopping trips. For the UK’s Higher Education system, this resulted in all universities closing their campuses, asking students to return home and moving to remote methods of teaching and assessment. These changes impacted an estimated 2.4 million students aged 18 years or older, and raised important questions about how they might be coping with the restrictions [2].

In particular, research has shown that being physically active is associated with a number of positive health outcomes including reduced mortality, improved musculoskeletal health, mental health and well-being and reduced levels of obesity [3,4,5]. A recent study found that individuals taking even small amounts of weekly exercise (e.g., 3–4 h per week) experienced 43% fewer days of poor mental health compared to those taking no exercise [6]. However, the COVID-19 pandemic has restricted PA in people of all ages, driven by the closure of gyms, public swimming pools, cancelation of team sports, reduced social interactions, and Government guidelines to leave the house only where necessary (although some exceptions were made for undertaking outdoor exercise) [7].

In the UK, a study of university students found that the COVID-19 pandemic resulted in a decrease in self-reported PA, especially in the first five weeks of lockdown [8]. Decreases in self-reported PA during the COVID-19 pandemic have also been reported among Italian university students [9], Spanish university students and young adults [10], Australian university students [11], US adults [12], and in an international survey [13]. Some of these studies also suggest that changes in PA might be modified by sociodemographic characteristics such as age and gender [7,8,10,11,14]. Indeed, prior evidence collected before the pandemic on PA levels of UK students has shown differences by gender, ethnicity and age [15].

However, several gaps in this research area remain. First, most studies exploring student PA during the pandemic have focused on self-reported questionnaires, which lack specificity and may introduce recall bias about the amount, type, and intensity of PA. The use of remote measurement technology (RMT) can offer a more accurate assessment of student PA, enabling continuous monitoring, whereas self-reported data tend to capture moments (snapshots) in time. RMT shows great promise in this regard, and can be an effective component of managing alcohol misuse [16,17], diet during pregnancy [18] and depression [19,20].

Second, studies to date have primarily focused on changes in PA before and after COVID-19 and the introduction of various restrictions. They have largely highlighted entirely predictable drops in PA between pre- and during lockdown, with short durations of follow-up. These drops likely reflect the temporary restrictions, uncertainty, caution and changes in motivation and commitment [21]. What is not known, and is perhaps of more long-term relevance, is how PA went on to change from the point of lockdown onwards, including subsequent periods when rules were relaxed but some social restrictions remained in place. Whether PA levels continued to worsen or whether they improved, and whether certain sociodemographic groups were particularly affected, could inform student health campaigns during these socially restrictive periods.

Finally, PA fluctuates over time, but previous studies in this area have conducted simple ‘before’ and ‘after’ comparisons rather than modelling PA trajectories. Powerful and intuitive longitudinal analysis techniques for repeated-measures data are available and commonly used in specialisms such as epidemiology [22,23]. Previous studies often approach the analysis of RMT data using data science approaches [20,24,25], whereas an epidemiological analytical approach could yield useful and informative insights. With PA during the COVID-19 pandemic being of significant interest to epidemiologists and data scientists alike, we took this opportunity to leverage methodological techniques from both disciplines, modelling RMT data using epidemiological analysis techniques.

The aims of this study were to describe trajectories of student PA following the start of UK lockdown and to investigate whether these trajectories varied according to key sociodemographic characteristics (age, gender and ethnicity). We hypothesised that PA would gradually increase over a period following the commencement of UK lockdown. This period encompasses the full lockdown period and some weeks of subsequent easing of restrictions, such that increases in PA might be expected due to changing attitudes towards restrictions, restriction easing, and the re-opening of leisure facilities. We also hypothesised that changes in PA would vary according to sociodemographic group, with groups known to be more vulnerable to the effects of coronavirus (males, older and ethnic minority groups) showing less pronounced increases in PA.

## 2. Materials and Methods

### 2.1. Ethical Permission

This study was undertaken in compliance with the Declaration of Helsinki. We used routinely collected anonymous data; ethical approval was obtained from the King’s College London Research Ethics Committee (reference: MRA-20/21-21871).

### 2.2. Design and Sample

This was a secondary, longitudinal and exploratory data analysis. The sample included all King’s College London (KCL) students who had enrolled in the RMT King’s Move PA tracker app [26] and had PA data available. King’s Move was launched 11 March 2020 and is available to all staff and students at KCL. The app collects and aggregates PA data from Google Fit and Apple Health fitness tracker apps and operates a rewards-based system: users collect ‘points’ for undertaking PA, which can then be redeemed for items such as gym passes, King’s Move branded merchandise, and food or drink from KCL cafes. User PA data are synced with KCL servers whenever a user interacts with the app, including prior activities.

For this study, data were extracted for a 12 week observation period from the commencement of UK lockdown on 23 March 2020. During the initial period of lockdown (7 weeks; 23 March to 10 May 2020), individuals could only leave the house for one form of exercise per day. In the second period of easing restrictions (5 weeks; 11 May to 14 June), individuals were able to return to work (where they could not work from home), spend time outdoors as often as they would like, and meet one other person from outside their own household [27].

Participants were eligible for inclusion in this study if they were a student at KCL and used the tracker app during the observation period. Users of the app who could not be linked with KCL student records, including their sociodemographic data, were ineligible, since these individuals likely represented staff members. Students who registered for King’s Move but did not use the tracker app (i.e., recorded no outcome data during the observation period) were also ineligible. We excluded participants who recorded PA data in the tracker app before the app had launched since these participants likely represented individuals developing or testing the app who could therefore introduce artificial PA data. The resulting eligible sample size was *n* = 770.

### 2.3. Measures

The outcome variables of interest were steps walked per week and miles run per week measured via the King’s Move tracker app. To reduce data noise, upper outliers for each outcome variable, defined as data points above the upper 95th percentile for the whole observation window, were removed.

The exposure variables of interest were weeks from lockdown commencement, gender, ethnicity and age (as of 20 August 2020). Week was specified as a continuous variable, with the week commencing 23 March 2020 denoted as Week 0, and the week commencing 8 June 2020 denoted as Week 11. Sociodemographic data were obtained from KCL student records. Due to small cell sizes, we were unable to model trajectories for all genders and ethnic groups. Therefore we dichotomised gender (male and female, not fitting trajectories for other gender identities), and ethnicity (White and all other ethnic groups combined). Please see limitations section for further information.

### 2.4. Statistical Analyses

To examine changes in PA following the start of lockdown, PA trajectories over the 12 weeks for each outcome were modelled using negative binomial mixed models for repeated measures. PA trajectories for each outcome were modelled with the main exposure variables as week, gender, ethnicity, and age. All exposures were included in the same model to control for each other’s effects. A random effect of user was included to account for correlated weekly PA within users. We specified an unstructured covariance matrix for the random effects of user, allowing all variances and covariances to be separately estimated. We used maximum likelihood estimation to include all available information (i.e., individuals with outcome information for at least one week were retained in the model, even if missing other weeks).

Since we do not assume a linear pattern of PA during and after lockdown, we tested for non-linear effects of week by adding quadratic and cubic terms to the model. Likelihood ratio tests were used to determine whether non-linear terms significantly improved model fit. To assess whether the resulting trajectories differed by sociodemographic characteristics, we included interaction terms between each sociodemographic characteristic and week, and again used likelihood ratio tests to examine whether they significantly improved model fit. We retained any statistically significant (*p* < 0.05) non-linear and sociodemographic interaction terms in the final models. We inspected plots to understand significant interaction terms. We report Incident Rate Ratios (IRR), 95% Confidence Intervals (CI), and *p*-values. All analyses were conducted in Stata 16.1.

### 2.5. Missing Data

Two participants were excluded because they had no PA data after removing outliers. A further *n* = 17 and *n* = 15 participants were omitted due to belonging to gender identities which were of too small a sample size to model trajectories, or due to missing ethnicity data, respectively. Therefore, our analytical sample comprised *n* = 736 participants, of which *n* = 730 had available data on the number of steps walked per week for at least one timepoint, and *n* = 264 had available data on the number of miles run per week for at least one timepoint. PA data availability for the outcome variables at each timepoint is shown in Appendix A Table A1.

## 3. Results

### 3.1. Descriptive Statistics

The majority of users in the analytical sample were female (*n* = 536, 72.8%), approximately half were from White ethnic groups (*n* = 393, 53.4%), and users had a median age of 22 years (IQR = 20–25). Excluded users were similar to the analytical sample in terms of gender, ethnicity and age (Table 1), although a slightly higher proportion of those included in running trajectory models were male and from White ethnic groups. Among those included in steps trajectory modelling (*n* = 730), the median number of steps walked each week during the observation period was 18,437 (IQR = 6970 to 34,607). Among those included in running trajectory modelling (*n* = 264), the median number of miles run per week during the observation period was 4.53 (IQR = 2.00 to 9.62).

### 3.2. Trajectories of Steps Walked per Week

A linear trajectory was initially fitted for steps walked per week, adjusted for gender, ethnicity and age. The addition of a quadratic effect for week significantly improved model fit (*X*^2^(1) = 3.97, *p* = 0.046) but a cubic effect for week did not further improve model fit (*X*^2^(2) = 4.36, *p* = 0.113). Fit of the quadratic model also did not improve with the further addition of interaction terms between gender and week (*X*^2^(2) = 1.85, *p* = 0.396), ethnicity and week (*X*^2^(2) = 1.60, *p* = 0.450), or age and week (*X*^2^(2) = 0.93, *p* = 0.629). Therefore, our final model included a quadratic effect of week and no interaction terms (Table 2).

**Figure 1:**
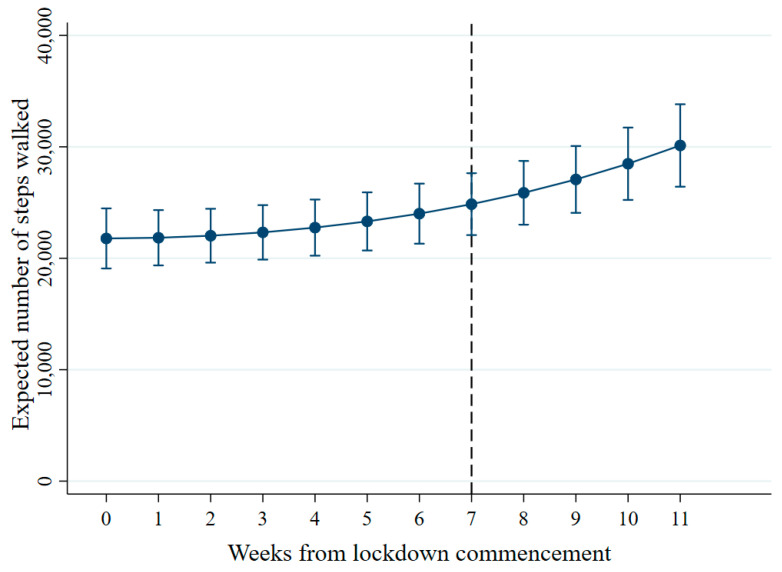
Trajectory of steps walked per week. The vertical dashed line indicates easing of lockdown restrictions (11 May 2020).

The results showed evidence for a gradual increase in PA over time on average, adjusting for other variables in the model (Figure 1). At Week 0, females recorded fewer steps walked compared to males (IRR = 0.79, 95% CI = 0.65 to 0.94, *p* = 0.010), and all other ethnic groups combined recorded fewer steps walked compared to White ethnic groups (IRR = 0.53, 95% CI = 0.45 to 0.62, *p* < 0.001). The fact that the above interaction terms between gender, ethnicity and week did not improve model fit suggested that these patterns were consistent over time.

### 3.3. Trajectories of Miles Run per Week

A linear trajectory was initially fitted for miles run per week, while adjusting for gender, ethnicity and age. Model fit was not significantly improved by the addition of a quadratic effect of week (*X*^2^(1) = 0.17, *p* = 0.681), and so further cubic effects were not fitted. Model fit was also not significantly improved by the addition of interaction terms between ethnicity and week (*X*^2^(1) = 0.50, *p* = 0.478) or age and week (*X*^2^(1) = 0.17, *p* = 0.682). However, the addition of an interaction term between gender and week did significantly improve model fit (*X*^2^(1) = 7.34, *p* = 0.007). Therefore, our final model included a linear effect of week, and an interaction term between gender and week (Table 3).

**Figure 2:**
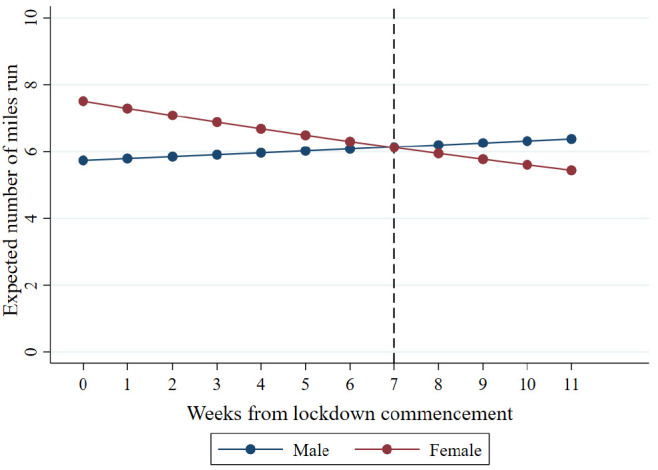
Trajectory of miles per week by gender. The vertical dashed lines indicate easing of lockdown restrictions (11 May 2020).

The results showed evidence for fewer miles run among all other ethnic groups combined compared to White ethnic groups at Week 0 (IRR = 0.57, 95% CI = 0.44 to 0.75, *p* < 0.001). The fact that the above interaction term between ethnicity and week did not improve model fit suggested that this pattern was consistent over time. However, the results did show evidence for an interaction between gender and week (IRR = 0.96, 95% CI = 0.94 to 0.99, *p* = 0.007). As shown in Figure 2, the resulting trajectories for each gender showed that females tended to show a decrease in the number of miles run per week during the observation period, whereas males did not.

## 4. Discussion

Our study took advantage of RMT data collected via the King’s Move PA tracker app, which launched just prior to the first UK nationwide lockdown, and epidemiological analysis techniques, to examine the PA trajectories of students at a leading Russell Group university in the UK. Our findings gave mixed support to our hypotheses and varied according to PA modality. In support of our first hypothesis, we found evidence for a gradual increase in the number of steps walked per week following the commencement of UK nationwide lockdown. However, there was no such evidence for an increase in the number of miles run. Furthermore, contrary to our second hypothesis, we did not find evidence for more vulnerable sociodemographic groups showing less pronounced increases in PA (although all other ethnic groups than White showed lower levels of PA throughout). Moreover, females showed a decrease in the number of miles run throughout the period, while males showed no such change.

Comparing PA before and during COVID-19 restrictions, previous studies have generally shown declines in PA among student populations [8,9,10,11]. We extended these findings by demonstrating an increase in steps walked from the commencement of lockdown onwards and subsequent easing of restrictions, as might be expected. This likely reflects increasing certainty and clarity over restrictions, and the gradual relaxation of rules permitting more outdoor activities and meeting friends [27]. Reductions in PA usually observed during lockdown may therefore be temporary [8,10,11,14], with students returning to higher levels of PA when permitted, although further research able to leverage pre-lockdown PA data would be needed to confirm this. This would be a positive finding, as PA is associated with long-term positive health outcomes, including mental well-being [6].

However, our finding that females showed a decrease in running behaviour following the commencement of lockdown is perhaps more surprising. Some previous studies found that PA among female students actually increased between pre- and during lockdown [14], particularly engaging in certain forms of exercise such as high-intensity interval training (known as HIIT) [10]. Compared to males, females more frequently reported that they had more time to exercise during confinement, that they had found different resources for exercising, and that exercise helped them against stress [10]. In light of this, it is plausible that our findings showing a decrease in running activity from during to post-lockdown might reflect female students having increased their exercise behaviour temporarily at the start of lockdown, but not maintaining this long term. Future studies could conduct qualitative work, and incorporate pre-lockdown PA data, to investigate whether this was indeed a temporary increase in running activity.

Other than these gender differences in running behaviour, we did not find changes in PA through lockdown and subsequent restriction easing to be modified by sociodemographic characteristics such as age or ethnicity. Nonetheless, for both walking and running outcome measures, we found that all other ethnic groups combined engaged in less PA than White ethnic groups throughout the observation period. This is in keeping with previous studies generally showing lower rates of PA among ethnic minority groups [28,29]. However, small sample sizes prevented us from exploring these differences using more granular ethnicity groupings. Future research drawing on larger samples should investigate ethnic differences in the impact of the pandemic on PA using more granular ethnic groupings, and should also consider other gender identities.

To our knowledge, this is one of the first studies to use RMT in plotting student PA trajectories from the commencement of UK lockdown. Existing studies have primarily relied on self-reported PA [8,11,14], introducing issues such as recall and reporting bias. Our study also demonstrates the feasibility of combining methodological techniques from data science and epidemiology disciplines to clearly analyse and display objective data collected from RMT devices.

### Strengths and Limitations

The current study is among the first to employ RMT to identify changes in PA in a UK university student sample during lockdown, without relying on self-reported ‘snapshot’ questionnaire data. This study contributes to the literature surrounding the impact of COVID-19 on PA in student cohorts [8,9,14]. This study was able to collect PA data via Google Fit and Apple Health, without reliance on self-reported questionnaires and the associated reporting biases [30]. However, there are several limitations.

First, users were linked to KCL student records via a student identification number for the purposes of extracting sociodemographic information. It was assumed that failed linkages and lack of sociodemographic information represented non-students, and potential linkage bias could not be investigated. Second, participants may have taken part in PA that was not captured by the King’s Move app, perhaps due to low device battery or a preferred PA modality not captured by the app. This could also be due to selection bias, with more physically active students willing to interact with the Kings Move app. Third, sample sizes were insufficient to conduct trajectory modelling for the number of miles cycled per week, with few students recording cycling data during the observation period. Finally, the King’s Move app was launched just prior to the UK nationwide lockdown, and it was not possible to ascertain whether PA changes were an artifact of increasing awareness of the King’s Move app. Our results are not necessarily generalisable to the King’s College London student community, or the wider student community. Future research will be undertaken to analyse PA using additional data collected from the King’s Move app.

## 5. Conclusions

In this study, we assessed changes in PA among students at a leading university in the UK during the national COVID-19 lockdown using RMT. Our findings demonstrate a mixed picture; we found an increase in the number of steps walked per week from the commencement of lockdown onwards, but a decrease in the number of miles run per week among female students. Our findings may help to develop and target interventions to improve PA and reduce sedentary behaviour during periods of lockdown.

## Figures and Tables

**Table 1:** Sociodemographic characteristics of student users excluded or included in analyses.

	Excluded	Analytical Sample	
	Student Users Excluded Due to Ineligibility or Missing Exposure Data (*n* = 265)	Sample for Steps Trajectory Modelling (*n* = 730)	Sample for Running Trajectory Modelling (*n* = 264)
Sociodemographic Characteristics	*n* (%)	*n* (%)	*n* (%)
Gender			
*Male*	71 (26.8%)	200 (27.4%)	95 (36.0%)
*Female*	174 (65.7%)	530 (72.6%)	169 (64.0%)
*Other*	20 (7.6%)	-	-
Ethnicity			
*White*	129 (48.7%)	388 (53.2%)	162 (61.4%)
*Black*	7 (2.6%)	24 (3.3%)	3 (1.1%)
*Asian*	77 (29.1%)	240 (32.9%)	77 (29.2%)
*Mixed*	21 (7.9%)	39 (5.3%)	11 (4.2%)
*Other*	15 (5.7%)	39 (5.3%)	11 (4.2%)
*Prefer not to say*	16 (6.0%)	-	-
Age in years as of 20 August 2020			
Median (IQR *)	22.0 (20.0 to 25.0)	22.0 (20.0 to 25.0)	22.5 (20.0 to 25.0)

* IQR: interquartile range.

**Table 2:** Fixed-effects parameter estimates from the final trajectory model for steps walked per week.

Fixed-Effects Parameter Estimates	IRR (95% CI)	*p*
**Estimated group differences in week 0**		
Gender		
*Male*	Reference	-
*Female*	0.79 (0.65 to 0.94)	0.010
Ethnicity		
*White*	Reference	-
*All other ethnic groups combined*	0.53 (0.45 to 0.62)	<0.001
Age	1.01 (1.00 to 1.03)	0.160
**Slope of outcome on week in the reference group**		
Linear effect	1.00 (0.97 to 1.03)	0.984
Quadratic effect	1.00 (1.00 to 1.01)	0.047

**Table 3:** Fixed-effects parameter estimates from the final trajectory model for miles run per week.

Fixed-Effects Parameter Estimates	IRR (95% CI)	*p*
**Estimated group differences in week 0**		
Gender		
*Male*	Reference	-
*Female*	1.31 (0.97 to 1.77)	0.082
Ethnicity		
*White*	Reference	-
*All other ethnic groups combined*	0.57 (0.44 to 0.75)	<0.001
Age	1.02 (1.00 to 1.05)	0.094
**Slope of outcome on week in the reference group**		
Linear effect	1.01 (0.99 to 1.03)	0.394
**Gender x week interaction**		
Female	0.96 (0.94 to 0.99)	0.007

## Data Availability

Researchers may apply to access a pseudonymised dataset. Requests to access study data are subject to submission of a research proposal to the corresponding author. All requests must be made in accordance with the UK Policy Framework for Health and Social Care research. Where the applicant is outside of King’s College London, a data-sharing agreement is required.

## Appendix A

**Table A1:** Availability of PA data at each timepoint.

	Steps Walked Per Week (*n* = 730)				Miles Run Per Week (*n* = 264)			
Timepoint	Missing		Observed		Missing		Observed	
	*n*	%	*n*	%	*n*	%	*n*	%
Week 0	427	58.5	303	41.5	194	73.5	70	26.5
Week 1	377	51.6	353	48.4	185	70.1	79	29.9
Week 2	418	57.3	312	42.7	168	63.6	96	36.4
Week 3	422	57.8	308	42.2	152	57.6	112	42.4
Week 4	434	59.5	296	40.5	160	60.6	104	39.4
Week 5	435	59.6	295	40.4	157	59.5	107	40.5
Week 6	449	61.5	281	38.5	172	65.2	92	34.8
Week 7	459	62.9	271	37.1	194	73.5	70	26.5
Week 8	456	62.5	274	37.5	177	67.0	87	33.0
Week 9	458	62.7	272	37.3	186	70.5	78	29.5
Week 10	448	61.4	282	38.6	194	73.5	70	26.5
Week 11	342	46.8	388	53.2	155	58.7	109	41.3
